# Estimated Reduction in Health Care Spending Associated With Weight Loss in Adults

**DOI:** 10.1001/jamanetworkopen.2024.49200

**Published:** 2024-12-05

**Authors:** Kenneth E. Thorpe, Peter J. Joski

**Affiliations:** 1 Department of Health Policy and Management, Rollins School of Public, Health Emory University, Atlanta, Georgia

## Abstract

**Importance:**

Recent data from federal surveys show that more than 70% of adults have either overweight or obesity. Overweight and obesity are associated with several expensive chronic conditions, such as type 2 diabetes, heart disease, hypertension, and hyperlipidemia. The prevalence of chronic disease is a key driver of rising health care spending among employers and the Medicare program.

**Objective:**

To estimate health care spending among adults with overweight or obesity and have employer-sponsored insurance or Medicare and examine the association of higher and lower body mass index (BMI).

**Design, Setting, and Participants:**

This cross-sectional study included privately insured adults and adult Medicare beneficiaries with a BMI of 25 and higher. Data from the Medical Expenditure Panel Survey–Household Component were used to tabulate total annual health care spending. Data were analyzed from April 1 to June 20, 2024.

**Main Outcomes and Measures:**

The primary intended measures were total annual health care spending, controlling for patient demographics, income, education, and BMI. The baseline health care spending as a percentage reduction in BMI. An instrumental variable regression model with estimated total spending at various levels of BMI was used.

**Results:**

This study included 3774 adults who were insured with Medicare (mean [SD] age, 63.1 [11.1] years; mean [SD] percentage female, 50.4% [49.7%]; mean [SD] percentage non-Hispanic Black adults, 17.4% [37.7%]; mean [SD] percentage non-Hispanic White adults, 61.3% [48.4%]). The study also included 13 435 adults who had employer-sponsored insurance (mean [SD] age, 46.3 [6.9] years; mean [SD] percentage female, 47.6% [48.9%]; mean [SD] percentage non-Hispanic Black adults, 11.1% [30.7%]; mean [SD] percentage non-Hispanic White adults, 73.1% [43.4%]). Overall, adults with employer-sponsored insurance with a weight loss of 5% were estimated to spend a mean of $670 (95% CI, $654-$686) less on health care (8% less), and those with a weight loss of 25% spent an estimated mean of $2849 (95% CI, $2783-$2916) less on health care (34% less). Among adults with Medicare who had 1 or more comorbid conditions, a 5% weight loss was estimated to reduce spending by $1262 (95% CI, $1217-$1306) (7% less) and a 25% weight loss was estimated to reduce health care spending by a mean of $5442 (95% CI, $5254-$5629) (31% less).

**Conclusions and Relevance:**

In this cross-sectional study, projected annual savings from weight loss among US adults with obesity were substantial for both Medicare and employer-based insurance.

## Key Points

**Question:**

What is the association of different levels of body mass index (BMI) and health care spending for adults with employer-sponsored insurance and Medicare?

**Findings:**

In this cross-sectional study of 17 209 adults, among those with employer-sponsored insurance, adults with a baseline BMI of 30 were estimated to spend 7% less following the loss of 5% of BMI and 30% less following the loss of 25% of BMI. Similar results were found for Medicare adults.

**Meaning:**

These findings suggest that a reduction in BMI among adults is associated with lower health care spending.

## Teaser

This cross-sectional study examines health care spending among adults who have overweight or obesity and evaluates changes in spending associated with different percentages of weight loss.

## Introduction

The share of US adults considered to have obesity, or a body mass index (BMI; calculated as weight in kilograms divided by height in meters squared) of 30 or more, has increased from 31% in 2000 to an estimated 42% in 2020. With the additional 31% of adults considered overweight,^1^ 73% of adults would need to lose weight to achieve recommended the BMI. Excess weight increases the likelihood of developing several chronic diseases, including type 2 diabetes, hypertension, heart diseases, hyperlipidemia, osteoarthritis, and pulmonary diseases, as well as increasing the risks of developing several types of cancer.

The rising prevalence of chronic disease over time, fueled in part by rising rates of overweight and obesity, is a major driver of the level and growth in health care spending. Recent estimates indicate that obesity is associated with more than $260 billion in annual health care spending in 2016 alone.^2^ With the anticipated growth in health spending, this total would rise to more than $385 billion in health spending in 2024.^3^

While most of the health care costs associated with obesity are paid by employer-based health insurance, Medicare and Medicaid shoulder approximately one-fourth of the costs, with consumers paying approximately 8% of the remaining spending out-of-pocket.^2^

Interventions to reduce the level and growth in obesity are major public health and economic opportunities with far-ranging benefits. Obesity has a major economic impact on employers because obesity in the workforce is associated with greater workplace accident rates and greater health care spending from employers and workers. Research on employer-related obesity costs found workers with obesity had almost twice as many workers’ compensation claims, 7 times higher medical claims costs, and 11 times greater indemnity claim costs compared with coworkers with a healthy weight (BMI, 18.5-24.9).^4^ Compared with other employees, workers with obesity required nearly 3 times more workers’ compensation days, 2.92 vs 8.59 days.^5^ Having a BMI greater than 35 increases the lifetime risk of developing type 2 diabetes to 70% or more,^6^ and every 5-point increase in BMI raises the risk of heart failure by nearly one-third.^7^ Moreover, excess weight gain accounts for 65% to 78% of the risks for developing hypertension.^8^ These data points provide a compelling rationale for all payers, both public and private, to invest in interventions to encourage the achievement and maintenance of healthy weight levels to reduce costs associated with obesity and related chronic conditions.

There are several evidence-based approaches to reduce the level and growth in obesity and overweight. Lifestyle intervention programs, such as the Diabetes Prevention Program, have been shown to be cost-effective (it is now a Medicare covered benefit) by producing a 5% to 7% reduction in weight.^9^ Bariatric surgery is another evidence-based option. Bariatric surgical procedures are expensive, involve inherent patient risks, and often require revisional surgical procedures or patients may not achieve and maintain weight loss. Payers, including Medicare, limit bariatric surgery to patients with a BMI of 35 or more and 1 obesity-related comorbid condition or a BMI of 40 or more.^10^ However, many studies examine the impact of bariatric surgery on health care spending. One recent meta-analysis found that weight loss resulting from bariatric surgery resulted in net health care savings.^11^

Finally, recent innovations in pharmacotherapy, such as GLP-1 agonist medications, provide a third option. These novel medications have been shown to produce substantial reductions in weight among people living with excess weight or obesity. At week 68, the mean difference among placebo and the GLP-1 tested groups was a 12% reduction in weight.^12^ However, approximately one-fourth of the adults using a GLP-1 agonist achieved a weight reduction of more than a 20%. While the costs of each of these interventions are well known, the reductions in health care spending for adults achieved through weight loss are less documented.

While some studies have estimated reductions in spending associated with various levels of weight loss, they have generally been limited to privately insured patients with a comorbid condition, such as diabetes, or limited to adults with private insurance. This study expands on this work by updating the results for reductions in health care spending associated with weight loss to 2023 dollars and includes reductions in health care spending in the Medicare program. In addition to looking at the mean association, we present estimates for the level of health care spending by baseline levels of BMI.

We examined changes in health care spending overall for those with 1 or more chronic conditions, including for 8 key chronic conditions associated with obesity: hyperlipidemia, hypertension, mental disorders, pulmonary disease, arthritis, back problems, heart disease, and asthma. The analysis presented changes in annual spending associated with 5%, 10%, 15%, 20%, and 25% reductions in baseline BMI.

## Methods

This cross-sectional study followed the Strengthening the Reporting of Observational Studies in Epidemiology (STROBE) reporting guideline. Because this study used deidentified survey data, institutional review board approval and informed consent were not required per the Common Rule.

We used data from the 2001 to 2016, 2018, and 2020 Medical Expenditure Panel Survey–Household Component (MEPS-HC) consolidated and medical condition data files.^11^ We used data from 2001 to 2016 and 2018 for adults with Medicare, and we used data from 2001 to 2016, 2018, and 2020 for adults with employer-sponsored insurance. We found some anomalies in the Medicare data during the COVID-19 pandemic in 2020. MEPS-HC is a nationally representative sample of the civilian noninstitutionalized population. It collects self-reported medical condition information, insurance coverage, health care use over 2 calendar years, and detailed demographics (eg, age, gender, race and ethnicity, and education). Because of the COVID-19 pandemic in 2020, the MEPS-HC primarily moved from in-person surveys to telephone interviews. The transition did not appear to affect reported BMI among those with private insurance, but did appear to affect those with Medicare.

Medical conditions in the MEPS-HC files are coded using Clinical Classifications Software (CCS) codes for years 2001 to 2015 and Clinical Classifications Software Refined (CCSR) codes for years after 2015. We selected respondents with 1 or more of the following 10 clinical conditions: diabetes (CCS 49, 50; CCSR END002-END006), hyperlipidemia (CCS 53; CCSR END010), hypertension (CCS 98, 99; CCSR CIR007 CIR008), mental health (CCS 650-663; CCSR MBD001-MBD014, MBD017-MBD034), pulmonary disease (CCS 127, 129-134; CCSR RSP006-RSP008, RSP010-RSP014, RSP016), arthritis (CCS 201-204; CCSR MUS00-MUS007), back problems (CCS 205; CCSR MUS011, MUS038), heart disease (CCS 96, 97, 100-108; CCSR CIR001-CIR006, CIR010-CIR012, CIR014-CIR018), cerebrovascular disease (CCS 109-113; CCSR NVS012, CIR020-CIR025) and asthma (CCS 128; CCSR RSP009). The analysis was limited to adults aged 24 to 64 years with a BMI of 25 or higher and 12 months of employer-sponsored insurance with a child in the household aged 11 to 20 years and adults aged 24 years or older with a BMI of 25 or higher and 12 months of Medicare with a child in the household aged 11 to 45 years. We excluded pregnant women and adults with a BMI greater than 80 from the analysis. We also excluded those missing weight data and other covariates in our regression model. Finally, we excluded adults with outlier expenditures above $506 000 (nominal) total expenditures. We estimated separate models for total health expenditures for those with employer-sponsored insurance and Medicare.

The survey estimation commands were used to adjust for the complex survey design of the MEPS-HC. All dollars were inflated to 2023 using the Personal Consumption Health Care Expenditures Index.^13^

### Statistical Analysis

There are several approaches that could be adopted to evaluate the association of weight and health care spending. These include microsimulation models (eg, Future Adult Model) and longitudinal analyses (eg, the work around the diabetes prevention program).^14,15^ Our analysis relied on cross-sectional data of health care spending and BMI. Following earlier previous work,^16^ we treated the BMI for each adult as an endogenous variable. Our analysis used a 2-stage residual inclusion instrumental variable model. Using the instrument strongly associated with BMI, the variable of interest, was critical to reliable estimation. In line with the approach by Cawley et al,^17^ we used the BMI of each adult’s oldest child. Instruments need to be highly associated with the endogenous variable and not associated with the error term in the second stage regression. For those with employer-sponsored insurance, we used the BMI of the oldest child between age 11 to 20 years as the instrument. For the Medicare population, we used the BMI of the oldest child from age 11 to 45 years. We used the associations mapping available in the MEPS-HC to link parents and children. The weight of a biological child has been shown to be a strong estimator of the parent’s weight because up to 75% of the variation in weight across individuals is genetic.^18^ Other work^2^ has shown the child’s BMI is a strong estimator of the parents BMI. The second requirement for an instrument is validity that the child’s BMI not be associated with the adult’s estimated medical spending after controlling for estimated BMI and other variables. However, a prior study^19^ found no measurable impacts of common experiences within the household and were largely irrelevant in determining individual differences in weight.

Generalized linear models (GLMs) with gamma distribution and log link function were estimated using total spending among those with at least 1 condition reported. The models controlled for BMI, race, gender, age groups, education, region of the country, marital status, household composition, self- or proxy-reported information, gender, age in months of oldest child whose BMI is used as instrument, and year. Race was included because it has been independently associated with BMI levels. Racial group included Asian, non-Hispanic Black, Hispanic, and non-Hispanic White. To get changes in health care spending, we estimated spending and then reduced BMI by 5%, 10%, 15%, 20%, and 25% to get estimated spending at the lower BMI levels We used 2-sided hypothesis tests with an a priori level of significance of *P* ≤ .05. Data were analyzed using Stata version 17 (StataCorp). Data were analyzed from April 1 to June 20, 2024.

## Results 

The study included 13 435 adults with employer-sponsored insurance (mean [SD] age, 46.3 [6.9] years; mean [SD] percentage female, 47.6% [48.9%]; mean [SD] percentage non-Hispanic Black adults, 11.1% [30.7%]; mean [SD] percentage non-Hispanic White adults, 73.1% [43.4%]). This study also included 3774 adults who were insured with Medicare (mean [SD] age, 63.1 [11.1] years; mean [SD] percentage female, 50.4% [49.7%]; mean [SD] percentage non-Hispanic Black adults, 17.4% [37.7%]; mean [SD] percentage non-Hispanic White adults, 61.3% [48.4%]). Summary baseline statistics for the variables used in the analysis were presented in Table 1 as well as in eTables 10 and 11 in Supplement 1.

**Table 1.** Baseline Population Characteristics of Adults With Employer-Sponsored Insurance and Medicare, BMI of 25 or Higher

Characteristic	Patients, mean (SD), %	
	Medicare (n = 3774)	Employer-sponsored insurance (n = 13 435)
Total spending, $	15 510 (31 253)	6913 (14 387)
BMI	32.5 (6.3)	31.5 (5.5)
Age, y	63.1 (11.1)	46.3 (6.9)
Oldest child BMI, mean (SD)	28 (7.5)	23.3 (5.1)
Oldest child female	41.6 (49.0)	47.8 (48.9)
Oldest child age, mo	363.6 (123.6)	195.1 (32.1)
Sex		
Female	50.4 (49.7)	47.6 (48.9)
Male	49.6 (49.7)	52.4 (48.9)
Race and ethnicity		
Hispanic	14 (34.5)	11 (30.6)
Non-Hispanic Asian	7.3 (25.8)	4.9 (21.1)
Non-Hispanic Black	17.4 (37.7)	11.1 (30.7)
Non-Hispanic White	61.3 (48.4)	73.1 (43.4)
Age category, y		
24-34	0.7 (8.4)	4.1 (19.4)
35-44	7.8 (26.7)	36.9 (47.2)
45-54	15.4 (35.9)	47.3 (48.8)
55-64	14.9 (35.4)	11.7 (31.4)
≥65	61.2 (48.4)	0
Education		
Less than high school	25.8 (43.5)	6.0 (23.2)
High school graduate	35.3 (47.5)	29.0 (44.4)
Some college	23.9 (42.4)	28.7 (44.2)
College graduate	15 (35.4)	36.3 (47.1)
Region		
Northeast	21.7 (41.0)	19.2 (38.5)
Midwest	19.7 (39.5)	25.2 (42.5)
South	39.4 (48.6)	36.5 (47.1)
West	19.2 (39.2)	19.1 (38.5)
Married	62.5 (48.1)	86.8 (33.1)
Self-reported information	61.8 (48.3)	55.9 (48.6)
No. in household age, y		
0-5	0.1 (0.3)	0.1 (0.4)
6-17	0.5 (0.9)	1.3 (1.0)
18-64	1.9 (1.0)	2.5 (0.8)
≥65	0.9 (0.8)	0 (0.2)
Family income as percentage of federal poverty line		
<100	12.8 (33.2)	1.5 (12.0)
100-199	24.9 (43.0)	9.6 (28.9)
200-399	31.6 (46.2)	35.8 (46.9)
≥400	30.7 (45.8)	53.0 (48.8)
Perceived health status		
Excellent	5.5 (22.6)	15.2 (35.1)
Very good	18.6 (38.7)	36.2 (47.0)
Good	34.6 (47.3)	36.4 (47.1)
Fair	27.9 (44.6)	10.0 (29.4)
Poor	13.4 (33.9)	2.3 (14.6)

Abbreviation: BMI, body mass index (calculated as weight in kilograms divided by height in meters squared).

### Employer-Sponsored Insured Results

For adults with a BMI of 25 or higher and employer-sponsored insurance, the mean (SD) total health care spending was $6913 ($14 387), and the mean (SD) BMI was 31.5 (5.5) (Table 1). The regression results of the employer-sponsored insurance adults are reported in eTable 1 in Supplement 1. We also reported the results from the second stage regression, the difference in baseline spending to the reduced BMI spending, and the difference between them. The residual term (Residual-ivbmi) of the first stage regression is included as a covariate (eTables 6-9 in Supplement 1).^20^ The *F* statistics in the first stage of the regressions were substantially higher in both models than the recommended threshold for minimum statistical power of an *F* of 10 or more (eTable 3 in Supplement 1). We show these first stage results in eTable 3 in Supplement 1. We provide an analysis that showed the instrument used was not associated with their parents spending in eTable 8 in Supplement 1. eAppendix 1 in Supplement 1 outlines the statistical methods for the instrumental variable analysis.

The association of BMI and health care spending among adults with employer-sponsored insurance was significant (*P* = .006). Each percentage point increase in BMI over 30 was associated with a mean increase in annual health care spending of $326 (95% CI, $93-$559). There were also large differences in annual spending by race and ethnicity. Relative to non-Hispanic White adults, Hispanic adults and non-Hispanic Black adults spent a mean of $2037 (95% CI, $1379-$2696) and $1548 (95% CI, $715-$2380) less per year, respectively.

Overall, adults achieving a 5% weight loss were estimated to spend a mean of $670 (95% CI, $654-$686) less on health care, representing a reduction of 8% (Table 2). The dollar and percentage reductions in spending increased sharply for each 5 percentage point reduction in weight. Adults with 25% weight loss were estimated to have spending reductions more than 4 times higher than those with a 5% weight loss. At a 25% weight loss, the estimated mean spending reduction was $2849 (95% CI, $2783-$2916), representing a reduction of 34%.

**Table 2.** Reduction in Annual Total Health Care Spending Per Condition, Adults Aged 24 to 64 Years With a BMI of 30 to 45 and Employer-Sponsored Health Insurance, 2023

Condition	Estimated baseline expenditures, mean (95% CI), $	Reductions in total health care spending by BMI reduction, mean (95% CI), $				
		5%	10%	15%	20%	25%
≥1 Conditions	8341 (8166 to 8516)	670 (654 to 686)	1286 (1256 to 1317)	1852 (1808 to 1896)	2372 (2316 to 2427)	2849 (2783 to 2916)
Diabetes	12 014 (11 435 to 12 593)	1840 (1738 to 1942)	3395 (3209 to 3581)	4709 (4454 to 4964)	5821 (5509 to 6133)	6761 (6402 to 7120)
Hyperlipidemia	8235 (7877 to 8593)	−200 (−209 to −192)	−406 (−424 to −388)	−616 (−644 to −589)	−832 (−869 to −795)	−1053 (−1100 to −1006)
Hypertension	8039 (7783 to 8294)	389 (376 to 403)	760 (734 to 786)	1112 (1074 to 1150)	1447 (1397 to 1497)	1765 (1705 to 1826)
Mental health	11 615 (11 175 to 12 055)	1289 (1233 to 1345)	2433 (2329 to 2538)	3449 (3302 to 3596)	4352 (4167 to 4536)	5153 (4936 to 5369)
Pulmonary disease (no asthma)	7802 (7390 to 8214)	−125 (−131 to −118)	−252 (−265 to −238)	−381 (−401 to −360)	−512 (−539 to −485)	−645 (−679 to −611)
Arthritis	13 249 (12 548 to 13 949)	1917 (1800 to 2034)	3553 (3339 to 3767)	4950 (4655 to 5244)	6143 (5781 to 6505)	7162 (6744 to 7581)
Back problems	11 775 (11 105 to 12 444)	1422 (1331 to 1513)	2670 (2501 to 2840)	3766 (3529 to 4004)	4729 (4433 to 5025)	5575 (5228 to 5921)
Heart and/or cerebrovascular disease	14 768 (13 712 to 15 824)	1034 (949 to 1119)	1995 (1832 to 2158)	2888 (2653 to 3122)	3717 (3417 to 4018)	4488 (4127 to 4849)
Pulmonary disease and/or asthma	8099 (7747 to 8452)	12 (11 to 12)	23 (22 to 24)	35 (33 to 36)	46 (44 to 48)	58 (55 to 60)

Abbreviation: BMI, body mass index (calculated as weight in kilograms divided by height in meters squared).

The results were similar for annual spending among patients with any 1 of the 9 chronic diseases but differed greatly among the individual conditions (Table 2). Projected reductions were greatest across all percentages of weight loss for people living with obesity and arthritis. A 15% weight loss resulted in estimated spending reductions that ranged from $1112 (95% CI, $1074-$1150) among those with hypertension to $4950 (95% CI, $4655-$5244) among adults with arthritis.

By the nature of the GLM model, the association of BMI and annual health care spending was nonlinear and varied by baseline BMI and percentage reduction in weight. These results were reported in eTable 4 in Supplement 1. Higher baseline BMI levels were associated with higher health care spending such that, the inverse: lower weight reduced health care spending held across all BMI and weight loss percentages. The potential for savings escalated according to baseline BMI such that the same percentage reduction in weight generated greater savings at higher BMI baseline levels. For instance, a 5% weight loss for an adult with baseline BMI of 30 was associated with a mean $441 (95% CI, $421-$461) reduction in spending, representing a reduction of 7%. The same 5% weight loss for an adult with a baseline BMI of 45 was associated with a mean of $1427 (95% CI, $1224-$1630) in annual health care costs, representing a reduction of 10% (eTable 4 in Supplement 1).

The reduction percentage in annual spending was most substantial among those with the highest BMI and largest weight loss percentage. For example, adults with 25% weight loss had a mean estimated reduction in spending that ranged from $1922 (95% CI, $1834-$2009) for adults with a baseline BMI of 30, representing a reduction of 30%, to $5824 (95% CI, $4996-$6654) among those with a BMI of 45, representing a 41% reduction (eTable 4 in Supplement 1).

### Medicare Results

Table 1 presented the baseline characteristics for Medicare enrollees with a BMI exceeding 24. Among Medicare adults with a BMI of 25 or more, the mean (SD) health care spending was $15 510 (31 253) and mean (SD) BMI was 32.5 (6.3).

The mean (SD) BMI of the oldest child used in the first-stage regressions was 28 (8). We used the same 2-stage least squares approach for Medicare adults as well. Each 1-point increase in BMI among Medicare adults was associated with a mean (SE) increase in total health care spending of $633 ($314) (eTable 2 in Supplement 1). Table 3 presents the reduction in annual health care spending associated with a 5% to 25% weight loss for adults with a BMI of 30 to 45. Adults with Medicare who had 1 or more comorbid condition who had 5% weight loss were estimated to reduce spending by a mean of $1262 (95% CI, $1217-$1306). The reduction in spending nearly doubled for a 10% weight loss for these individuals, with a mean reduction in spending of $2430 (95% CI, $2344-$2515). For 25% weight loss, the reduction in spending doubled again (relative to the 10% reduction), and spending was cut by a mean of $5442 (95% CI, $5254-$5629) compared with spending at the baseline weight, representing a 31% reduction in health care costs (Table 3). Among Medicare beneficiaries with 1 or more comorbid conditions who had a 15% reduction in BMI, the estimated spending decreased by a mean of $3512 (95% CI, $3389-$3634) from baseline, representing a 20% reduction in spending.

**Table 3.** Reduction in Annual Total Health Care Spending Per Condition, Adults (Aged 24 Years or Older), BMI of 30 to 45, and Medicare, 2023

Condition	Estimateed baseline expenditure, mean (95% CI), $	Reductions in total health care spending by BMI reduction, mean (95% CI), $				
		5%	10%	15%	20%	25%
≥1 Conditions	17 284 (16 758 to 17 810)	1262 (1217 to 1306)	2430 (2344 to 2515)	3511 (3389 to 3634)	4513 (4357 to 4670)	5442 (5254 to 5629)
Diabetes	19 899 (19 011 to 20 787)	651 (619 to 684)	1281 (1217 to 1345)	1890 (1795 to 1984)	2478 (2355 to 2602)	3047 (2896 to 3199)
Hyperlipidemia	17 167 (16 304 to 18 029)	913 (861 to 965)	1776 (1675 to 1877)	2593 (2447 to 2739)	3366 (3176 to 3555)	4097 (3867 to 4327)
Hypertension	18 447 (17 655 to 19 238)	1331 (1266 to 1397)	2565 (2439 to 2692)	3709 (3528 to 3891)	4769 (4537 to 5001)	5752 (5474 to 6031)
Mental health	20 923 (19 797 to 22 049)	438 (414 to 461)	866 (819 to 912)	1285 (1216 to 1354)	1695 (1604 to 1786)	2097 (1984 to 2209)
Pulmonary disease (no asthma)	20 671 (19 366 to 21 976)	771 (721 to 821)	1513 (1416 to 1611)	2227 (2084 to 2370)	2914 (2727 to 3101)	3575 (3345 to 3805)
Arthritis	18 575 (17 760 to 19 391)	572 (545 to 598)	1126 (1074 to 1178)	1662 (1586 to 1739)	2182 (2082 to 2283)	2686 (2562 to 2810)
Back problems	18 087 (16 774 to 19 400)	-388 (-417 to -360)	-785 (-843 to -727)	−1191 (−1279 to −1102)	−1605 (−1724 to −1486)	−2028 (−2179 to −1877)
Heart and/or cerebrovascular disease	21 076 (19 526 to 22 626)	−369 (−397 to −342)	−746 (−801 to −691)	−1128 (−1212 to −1045)	−1518 (−1630 to −1406)	−1914 (−2056 to −1773)
Pulmonary disease and/or asthma	20 135 (19 049 to 21 221)	644 (608 to 680)	1267 (1196 to 1338)	1870 (1766 to 1975)	2454 (2317 to 2591)	3018 (2850 to 3187)

Abbreviation: BMI, body mass index (calculated as weight in kilograms divided by height in meters squared).

As displayed in eTable 5 in Supplement 1, the dollar and percentage reductions in health spending for a given reduction percentage in BMI were far greater for higher baseline BMIs. Following the same pattern reported for those with employer-sponsored insurance, for a given percentage reduction in weight increases in baseline BMI resulted in larger dollar and percent reductions in health care spending. For example, among adults with Medicare with a baseline BMI of 30, 15% weight loss was associated with a mean reduction in spending of $2352 (95% CI, $2206-$2497), representing a 17% reduction in spending, and 25% weight loss was associated with a mean reduction in health care spending of $3686 (95% CI, $3458-$3915), representing a reduction of 27% (eTable 5 in Supplement 1). At the extreme, an adult with a baseline BMI of 45 with a 15% weight loss was associated with a mean reduction in spending of $6271 (95% CI, $5204-$7339), representing a spending reduction of 25%, and 25% weight loss was associated with a mean reduction in spending of $9557 (95% CI, $7930-$11 184), representing a spending reduction of 38% (eTable 5 in Supplement 1).

The results were similar for annual spending among Medicare beneficiaries with baseline BMI of 30 or more and having any 1 of the 9 chronic diseases, although the magnitude of savings varied by conditions. Projected savings were high across all weight loss percentages for individuals with obesity and hypertension. Weight loss of 15% resulted in estimated mean spending reductions that ranged from $1285 (95% CI, $1216-$1354), representing a spending reduction of 6% among those with mental health disease to $3709 (95% CI, $3528-$3891) among those with hypertension, representing a spending reduction of 20% (Table 3).

## Discussion

The findings of this study showed the reduction in estimated health care spending was associated with weight loss. This study updated previous work and expanded it to include the impact of weight loss on Medicare adults. Our results showed that changes in the distribution of BMI among adults can produce substantial reductions in health care spending.

The approaches for generating weight loss in these ranges are currently available. Randomized clinical trials of the use of GLP-1 antagonists have found that more than a one-third of participating adults with overweight or obesity who used the product lost 20% or more of their body weight. On average, adults lost nearly 13% of body weight. Given the substantial projected reduction in health care spending associated with the weight loss among people with obesity, these results may be of special interest to employers, health plans, and Medicare. Medicare does not currently reimburse for weight loss medications approved by the US Food and Drug Administration, which may be a missed opportunity. Many employers also deny coverage outright or significantly limit coverage of the drugs. This analysis of the projected reduction in health care spending associated with weight loss can provide important information for changes in policy toward reimbursement for evidence-based weight loss treatments.

### Limitations

This study has limitations. These results are based on a cross-sectional analysis rather than a longitudinal analysis. This study does not track patients over time printerevention and postintervention, rather it looks at data from one point in time, which was a limitation.

## Conclusions

The projected annual savings from weight loss among US adults with obesity were considerable for both Medicare and employer-based insurance. Projected savings started at modest levels of weight loss and accelerated by multiples as weight loss percentage increased. Projected savings were consistent for employers and Medicare and were highest for people with higher baseline BMI levels. The common presence of comorbid chronic conditions associated with having overweight or obesity were estimated to have savings from weight reduction across BMI levels of 30 or more, and those with type 2 diabetes had the highest level of estimated savings. However, these savings did not reflect the potential of promoting weight loss to prevent the development of common chronic conditions associated with excess weight and obesity. Those conditions, including diabetes, heart disease, stroke, hypertension, and many cancers, are primary drivers of health care spending, preventable disability, economic losses, and premature death. Accordingly, greater overall benefits could be realized by Medicare, private insurers, individuals, and society at large from promoting weight loss and healthy weight maintenance among people living with excess weight or obesity. Achieving weight loss and the projected savings was facilitated by improving access to evidence-based programs and treatments demonstrated to promote and maintain weight loss. Improving access to new weight loss medications, along with existing evidence-based behavior change and weight loss interventions, should help reduce health care spending associated with obesity in the US.

## Notes

Supplement 1. **eAppendix 1.** Statistical Methods for the Instrumental Variable Analysis **eTable 1.** Second Stage Regression Results Adults With Employer-Sponsored Insurance **eTable 2.** Second Stage Regression Results for Adults With Medicare **eTable 3.** Supplemental Regressions: F Statistics From First Stage of Instrumental Variable Regression **eTable 4.** Estimated Change in Health Care Spending by Percent BMI Reduction, Adults With Employer-Sponsored Insurance, Estimated Spending and Standard Errors **eTable 5.** Estimated Change in Health Care Spending by Percent BMI Reductions, Adults With Medicare, Estimated Spending and Standard Errors **eTable 6.** Association Between Adult BMI and BMI of Oldest Child Between Ages of 11 to 20 Years for Adults With Employer-Sponsored Insurance **eTable 7.** Association Between Adult BMI and BMI of Oldest Child Between Ages of 11 to 45 Years for Adults With Medicare **eTable 8.** Association Between Adult Health Care Expenditures and BMI of Oldest Child Between Ages of 11 to 20 Years for Adults With Employer-Sponsored Insurance **eTable 9.** Association Between Adult Health Care Expenditures and BMI of Oldest Child Between Ages of 11 to 45 Years for Adults With Medicare **eTable 10.** Patient Characteristics by BMI of Oldest Child Between Ages of 11 to 20 Years for Adults With Employer-Sponsored Insurance **eTable 11.** Patient Characteristics by BMI of Oldest Child Between Ages of 11 to 45 Years for Adults With Medicare

Supplement 2.
Data Sharing Statement
