# Supplementary material for: Estimated Reduction in Health Care Spending Associated With Weight Loss in Adults
Source: JAMA Netw Open. 2024 Dec 5;7(12):e2449200. doi: 10.1001/jamanetworkopen.2024.49200 (PMC11621981; doi:10.1001/jamanetworkopen.2024.49200)
Supplement: Supplement 1. — eAppendix 1. Statistical Methods for the Instrumental Variable Analysis eTable 1. Second Stage Regression Results Adults With Employer-Sponsored Insurance eTable 2. Second Stage Regression Results for Adults With Medicare eTable 3. Supplemental Regressions: F Statistics From First Stage of Instrumental Variable Regression eTable 4. Estimated Change in Health Care Spending by Percent BMI Reduction, Adults With Employer-Sponsored Insurance, Estimated Spending and Standard Errors eTable 5. Estimated Change in Health Care Spending by Percent BMI Reductions, Adults With Medicare, Estimated Spending and Standard Errors eTable 6. Association Between Adult BMI and BMI of Oldest Child Between Ages of 11 to 20 Years for Adults With Employer-Sponsored Insurance eTable 7. Association Between Adult BMI and BMI of Oldest Child Between Ages of 11 to 45 Years for Adults With Medicare eTable 8. Association Between Adult Health Care Expenditures and BMI of Oldest Child Between Ages of 11 to 20 Years for Adults With Employer-Sponsored Insurance eTable 9. Association Between Adult Health Care Expenditures and BMI of Oldest Child Between Ages of 11 to 45 Years for Adults With Medicare eTable 10. Patient Characteristics by BMI of Oldest Child Between Ages of 11 to 20 Years for Adults With Employer-Sponsored Insurance eTable 11. Patient Characteristics by BMI of Oldest Child Between Ages of 11 to 45 Years for Adults With Medicare [file jamanetwopen-e2449200-s001.pdf]

Supplemental Online Content

Thorpe KE, Joski PJ. Estimated reduction in health care spending associated with weight loss among adults. *JAMA Netw Open*. 2024;7(12):e2449200.
doi:10.1001/jamanetworkopen.2024.49200

eAppendix 1. Statistical Methods for the Instrumental Variable Analysis

eTable 1. Second Stage Regression Results Adults With Employer-Sponsored Insurance

eTable 2. Second Stage Regression Results for Adults With Medicare

eTable 3. Supplemental Regressions: F Statistics From First Stage of Instrumental Variable Regression

eTable 4. Estimated Change in Health Care Spending by Percent BMI Reduction, Adults With Employer-Sponsored Insurance, Estimated Spending and Standard Errors

eTable 5. Estimated Change in Health Care Spending by Percent BMI Reductions, Adults With Medicare, Estimated Spending and Standard Errors

eTable 6. Association Between Adult BMI and BMI of Oldest Child Between Ages of 11 to 20 Years for Adults With Employer-Sponsored Insurance

eTable 7. Association Between Adult BMI and BMI of Oldest Child Between Ages of 11 to 45 Years for Adults With Medicare

eTable 8. Association Between Adult Health Care Expenditures and BMI of Oldest Child Between Ages of 11 to 20 Years for Adults With Employer-Sponsored Insurance

eTable 9. Association Between Adult Health Care Expenditures and BMI of Oldest Child Between Ages of 11 to 45 Years for Adults With Medicare

eTable 10. Patient Characteristics by BMI of Oldest Child Between Ages of 11 to 20 Years for Adults With Employer-Sponsored Insurance

eTable 11. Patient Characteristics by BMI of Oldest Child Between Ages of 11 to 45 Years for adults with Medicare

This supplemental material has been provided by the authors to give readers additional information about their work.

© 2024 Thorpe KE et al. *JAMA Network Open*.

eAppendix 1. Statistical methods for the instrumental variable analysis

We used the BMI of the parents' child as the instrument in the analysis. Child BMI has been used as an instrument for their parents in previously published work.

In our model, the first stage using the child's BMI had F- statistics ranging from 19 to nearly 295 in the employer model and 23 to 109 for the Medicare model. For each of the conditions and total spending, the first stage of each model had F-statistics that far exceeded the minimum standard showing significant power. We showed these first stage results in eTable 3 in Supplement 1.

There are three requirements for an instrument to be an IV. First, it must be a powerful predictor of the endogenous variable, parents BMI in this case. Second, the instrument must be uncorrelated with the error term in the second stage regression. In this case, the weight of the child must not be correlated with the parents health care spending after controlling for the predicted BMI and other covariates. Third, there should be no confounders between the instrument and the outcome. We tested for the first two conditions and did an analysis of the third.

Condition 1. We report the F-statistics of the first stage regression in eTable 3. The statistical rule of thumb is that an instrument is a powerful predictor if it has an F-Statistic >10. For the employer-sponsored insurance model the F-Statistics ranged from 19 to 295 and in the Medicare model from 23 to 109. These are indicative that the child's BMI had significant power.

Condition 2. The instrument must not be associated with the outcome, parents health care spending. In our adjusted analysis a child's BMI was not correlated with parents health care spending with a p-value of 0.19 for adults with employer-sponsored insurance (eTable 8) and a p-value of 0.25 for adults with Medicare (eTable 9).

Condition 3. We examined whether the effect of child BMI on parents spending was confounded by other measures such as economic status and health status. To evaluate this, we examined the association between categories of child BMI (< 30 and ≥ 30) and the potential confounding variables as reported in eTables 10 and 11. We examined whether the observed confounders were comparable (balanced) across categories of the child's BMI.

Starting with measures of social economic status such as education there were no significant differences across categories of child BMI. There were also no significant differences across the categories by race.

© 2024 Thorpe KE et al. *JAMA Network Open*.

eTable 1. Second Stage Regression Results Adults with Employer-Sponsored Insurance

Average marginal effects

	dy/dx	Linearized std. err.	t	P>|t|	[95% conf.	interval]
Residual-ivbmi	-262.2859	119.7487	-2.19	0.029	-497.3415	-27.23026
Adult bmi	326.2447	118.8095	2.75	0.006	93.03262	559.4568
female	1439.998	307.3466	4.69	0.000	836.7048	2043.291
Race/ethnicity						
Non-Hisp Black	-1547.69	424.0912	-3.65	0.000	-2380.142	-715.238
Non-Hisp Other	-527.2508	600.3681	-0.88	0.380	-1705.718	651.2166
Hispanic	-2037.246	335.4827	-6.07	0.000	-2695.768	-1378.724
Age Categories						
35-44	-142.8953	640.5881	-0.22	0.824	-1400.311	1114.52
45-54	690.4588	660.0031	1.05	0.296	-605.0665	1985.984
55-64	1917.01	800.5496	2.39	0.017	345.6045	3488.415
Education						
<HS_grad	350.4504	511.9263	0.68	0.494	-654.4138	1355.315
Some_College	891.4933	382.8977	2.33	0.020	139.9004	1643.086
College_grad	963.3644	386.4116	2.49	0.013	204.874	1721.855
Region						
midwest	-123.7717	462.1163	-0.27	0.789	-1030.864	783.3201
south	-897.824	430.3569	-2.09	0.037	-1742.575	-53.07299
west	-454.9035	490.0427	-0.93	0.354	-1416.812	507.0053
married	1372.69	429.8665	3.19	0.001	528.9019	2216.479
Number in Household						
Ages 0-5	-744.9778	317.8562	-2.34	0.019	-1368.9	-121.0554
Ages 6-17	-329.7609	159.82	-2.06	0.039	-643.4728	-16.04902
Ages 18-64	-299.9972	232.3448	-1.29	0.197	-756.0686	156.0742
Ages 65+	-334.7721	730.4859	-0.46	0.647	-1768.649	1099.104
Self report	488.6903	323.3856	1.51	0.131	-146.0859	1123.467
year						
2002	666.3019	418.9516	1.59	0.112	-156.0615	1488.665
2003	218.8914	412.4818	0.53	0.596	-590.7724	1028.555
2004	1446.78	565.7625	2.56	0.011	336.2404	2557.32
2005	510.6819	504.006	1.01	0.311	-478.6356	1499.999
2006	447.7822	515.7754	0.87	0.386	-564.6374	1460.202
2007	1325.507	865.7154	1.53	0.126	-373.8128	3024.826
2008	785.3864	614.2257	1.28	0.201	-420.2822	1991.055
2009	972.606	495.6023	1.96	0.050	-.2157587	1945.428
2010	1534.625	619.5844	2.48	0.013	318.4381	2750.812
2011	843.6039	643.103	1.31	0.190	-418.748	2105.956
2012	7.188818	518.3742	0.01	0.989	-1010.332	1024.71
2013	1463.042	579.6425	2.52	0.012	325.2576	2600.827
2014	751.0002	675.1981	1.11	0.266	-574.3514	2076.352
2015	109.3419	669.4359	0.16	0.870	-1204.699	1423.383
2016	2708.048	934.8259	2.90	0.004	873.0706	4543.025
2018	3038.368	874.9102	3.47	0.001	1321	4755.736
2020	4079.759	1686.337	2.42	0.016	769.634	7389.883
Child female	521.9906	262.7259	1.99	0.047	6.283849	1037.697
Child age	.6406741	5.585566	0.11	0.909	-10.32328	11.60463

© 2024 Thorpe KE et al. *JAMA Network Open*.

```
Family income as percent of poverty line
 100-199 FPL  |  -665.4107   1163.868    -0.57   0.568    -2949.976    1619.155
 200-399 FPL  |  -1358.019   1099.306    -1.24   0.217    -3515.856    799.8185
    400+ FPL  |  -1124.351   1122.708    -1.00   0.317    -3328.123    1079.421
              |
Health status |
    verygood  |   801.0758   267.9656     2.99   0.003      275.084    1327.068
        good  |   2360.511   368.1791     6.41   0.000      1637.81    3083.213
        fair  |     6998.8   816.1989     8.57   0.000     5396.677    8600.924
        poor  |   16154.93   2536.373     6.37   0.000     11176.26    21133.59
------------------------------------------------------------------------------
Note: dy/dx for factor levels is the discrete change from the base level.

.
```

© 2024 Thorpe KE et al. *JAMA Network Open*.

eTable 2. Second Stage Regression Results for Adults with Medicare

Average marginal effects

	dy/dx	Linearized std. err.	t	P>|t|	[95% conf.	interval]
Residual-ivbmi	-541.0339	315.9426	-1.71	0.088	-1162.126	80.05827
Adult bmi	633.1073	314.995	2.01	0.045	13.87793	1252.337
female	-2473.115	1235.435	-2.00	0.046	-4901.781	-44.44875
Race/ethnicity						
Non-Hisp Black	-1201.172	1130.946	-1.06	0.289	-3424.429	1022.086
Non-Hisp Other	-374.1942	2092.471	-0.18	0.858	-4487.654	3739.266
Hispanic	-140.206	1311.032	-0.11	0.915	-2717.483	2437.071
Age Categories						
35-44	4560.264	2749.501	1.66	0.098	-844.8115	9965.339
45-54	5319.46	2716.585	1.96	0.051	-20.90809	10659.83
55-64	4450.071	2612.199	1.70	0.089	-685.0898	9585.232
65+	2118.705	2859.551	0.74	0.459	-3502.71	7740.121
Education						
<HS_grad	-1290.293	976.2431	-1.32	0.187	-3209.429	628.8439
Some_College	2610.409	1209.092	2.16	0.031	233.5284	4987.289
College_grad	4234.024	1994.374	2.12	0.034	313.4055	8154.642
region						
Midwest	-2079.165	1596.494	-1.30	0.194	-5217.615	1059.285
South	-1999.128	1340.819	-1.49	0.137	-4634.961	636.7047
West	-2005.454	1614.373	-1.24	0.215	-5179.051	1168.144
married	-1641.394	1254.178	-1.31	0.191	-4106.905	824.1173
Number in Household						
Ages 0-5	-2122.602	1136.891	-1.87	0.063	-4357.545	112.3422
Ages 6-17	-581.5481	607.8538	-0.96	0.339	-1776.491	613.3945
Ages 18-64	59.26983	578.771	0.10	0.918	-1078.501	1197.04
Ages 65+	-1235.315	1241.588	-0.99	0.320	-3676.077	1205.447
self report	750.6484	958.4267	0.78	0.434	-1133.464	2634.761
year						
2002	826.1914	3250.193	0.25	0.799	-5563.163	7215.546
2003	-2413.055	3025.318	-0.80	0.426	-8360.342	3534.232
2004	-3401.129	2368.336	-1.44	0.152	-8056.896	1254.638
2005	-2813.851	2671.267	-1.05	0.293	-8065.131	2437.429
2006	448.3936	2981.241	0.15	0.881	-5412.246	6309.033
2007	-732.338	2730.593	-0.27	0.789	-6100.243	4635.568
2008	-1388.932	2811.951	-0.49	0.622	-6916.774	4138.91
2009	-2887.517	2573.585	-1.12	0.263	-7946.77	2171.735
2010	-1182.152	2724.544	-0.43	0.665	-6538.166	4173.862
2011	-3159.848	2524.233	-1.25	0.211	-8122.082	1802.386
2012	-2357.663	2544.427	-0.93	0.355	-7359.595	2644.27
2013	-183.1661	2832.385	-0.06	0.948	-5751.177	5384.845
2014	632.601	2833.74	0.22	0.823	-4938.076	6203.278
2015	265.331	2845.725	0.09	0.926	-5328.906	5859.568
2016	-2478.173	2673.834	-0.93	0.355	-7734.5	2778.153
2018	1598.129	2878.187	0.56	0.579	-4059.923	7256.181
child female	1136.418	865.8935	1.31	0.190	-565.7889	2838.625
Child age	7.898415	5.622306	1.40	0.161	-3.154132	18.95096
Family income as percent of poverty line						
100-199 FPL	841.2315	1443.16	0.58	0.560	-1995.788	3678.251

© 2024 Thorpe KE et al. *JAMA Network Open*.

```
      200-399 FPL |  -456.7089    1388.18    -0.33   0.742    -3185.647    2272.229
         400+ FPL |   675.2928   1835.636     0.37   0.713    -2933.271    4283.857
                  |
    Health status |
        Very good |    2686.27   852.3046     3.15   0.002     1010.776    4361.763
            good  |    5943.44   981.4492     6.06   0.000      4014.07    7872.811
            fair  |   11092.79   1323.938     8.38   0.000     8490.141    13695.44
            poor  |   16840.22   1903.633     8.85   0.000     13097.98    20582.46
------------------------------------------------------------------------------------
Note: dy/dx for factor levels is the discrete change from the base level.
```

© 2024 Thorpe KE et al. *JAMA Network Open*.

eTable 3. Supplemental Regressions: *F* Statistics from First Stage of Instrumental Variable Regression

Employer-Sponsored Insurance Aged (24-64) with child 11-20, BMI>=25

First Stage Instrumental Variable Regression Results<br>Instrument: BMI of Oldest Child between 11 to 20 Years Old<br>Employer Sponsored Insurance		
**Condition**	**Partial R2**	**F – Statistic (P-Value)**
>=1 Condition	0.0389	294.96 (<0.001)
Diabetes	0.0144	19.17 (<0.001)
Hyperlipidemia	0.0343	71.65 (<0.001)
Hypertension	0.0446	143.76 (<0.001)
Mental Health Disorders	0.0427	81.24 (<0.001)
Pulmonary Disease	0.0320	54.12 (<0.001)
Arthritis	0.0439	53.91 (<0.001)
Back Problems	0.0378	36.71 (<0.001)
Heart or Cerebrovascular Disease	0.0225	19.92 (<0.001)
Pulmonary disease or asthma	0.0342	70.11 (<0.001)

Medicare Aged (24+) with child 11-45, BMI>=25

First Stage Instrumental Variable Regression Results<br>Instrument: BMI of Oldest Child between 11 to 45 Years Old<br>Medicare		
**Condition**	**Partial R2**	**F – Statistic (P-Value)**
>=1 Condition	0.0513	108.97 (<0.001)
Diabetes	0.0422	32.51 (<0.001)
Hyperlipidemia	0.0423	40.01 (<0.001)
Hypertension	0.0567	79.59 (<0.001)
Mental Health Disorders	0.0504	32.59 (<0.001)
Pulmonary Disease	0.0555	26.55 (<0.001)
Arthritis	0.0592	65.27 (<0.001)
Back Problems	0.0690	26.90 (<0.001)
Heart or Cerebrovascular Disease	0.0347	22.86 (<0.001)
Pulmonary disease or asthma	0.0519	29.73 (<0.001)

© 2024 Thorpe KE et al. *JAMA Network Open*.

eTable 4. Estimated Change in Health Care Spending by Percent BMI Reduction, Adults with Employer-Sponsored Insurance , Predicted Spending and Standard Errors

		BMI REDUCTION AND REDUCTION IN SPENDING				
Adult BMI	PREDICTED Spending	5%	10%	15%	20%	25%
30	6380.93 (3270.97)	441.31 (226.51)	852.09 (437.33)	1234.46 (633.55)	1590.39 (816.18)	1921.69 (986.16)
31	6750.68 (3309.92)	482.29 (236.65)	930.11 (456.38)	1345.94 (660.39)	1732.07 (849.81)	2090.60 (1025.68)
32	7010.08 (3584.61)	516.29 (264.35)	994.56 (509.20)	1437.59 (736.00)	1848.00 (946.06)	2228.18 (1140.63)
33	7340.26 (3632.32)	556.65 (275.71)	1071.08 (530.49)	1546.49 (765.93)	1985.86 (983.49)	2391.90 (1184.53)
34	8288.25 (4571.03)	645.95 (356.06)	1241.55 (684.38)	1790.73 (987.13)	2297.11 (1266.29)	2764.03 (1523.70)
35	8448.60 (3944.27)	676.87 (316.19)	1299.50 (607.03)	1872.26 (874.55)	2399.12 (1120.62)	2883.77 (1346.96)
36	9270.83 (4694.85)	762.99 (386.35)	1463.18 (740.90)	2105.75 (1066.27)	2695.43 (1364.86)	3236.57 (1638.87)
37	9655.64 (4688.78)	816.65 (397.99)	1564.23 (762.19)	2248.58 (1095.48)	2875.05 (1400.48)	3448.52 (1679.58)
38	10602.57 (5482.33)	917.44 (475.11)	1755.49 (909.03)	2521.02 (1305.35)	3220.31 (1667.32)	3859.08 (1997.92)
39	10520.88 (5269.29)	934.38 (468.08)	1785.78 (894.57)	2561.56 (1283.17)	3268.43 (1637.25)	3912.53 (1959.87)
40	11679.79 (6169.65)	1063.33 (562.32)	2029.85 (1073.38)	2908.37 (1537.86)	3706.91 (1960)	4432.75 (2343.66)
41	11785.68 (6255.22)	1096.67 (578.62)	2091.29 (1103.71)	2993.36 (1580.23)	3811.48 (2012.66)	4553.48 (2405.09)
42	12850.96 (6720.04)	1222.96 (639.38)	2329.54 (1217.93)	3330.81 (1741.42)	4236.78 (2215.10)	5056.54 (2643.71)
43	13075.21 (6399.58)	1274.13 (626.31)	2424.10 (1191.33)	3462.01 (1701.04)	4398.77 (2160.86)	5244.24 (2575.69)
44	14980.32 (7635.70)	1490.91 (758.43)	2833.43 (1441.53)	4042.33 (2056.77)	5130.92 (2610.89)	6111.16 (3109.97)
45	14040.64 (7901.82)	1426.96 (803.35)	2708.90 (1525.01)	3860.55 (2173.30)	4895.15 (2755.68)	5824.60 (3278.85)
Total	8341.21 (4821.67)	670.35 (439.95)	1286.16 (839.01)	1851.92 (1201.06)	2371.75 (1529.60)	2849.43 (1827.80)

© 2024 Thorpe KE et al. *JAMA Network Open*.

eTable 5. Estimated Change in Health Care Spending by Percent BMI Reductions, Adults with Medicare, Estimated Spending and Standard Errors

		BMI REDUCTION AND REDUCTION IN SPENDING				
Adult BMI	Predicted Spending	5%	10%	15%	20%	25%
30	13448.89 (5862.99)	834.63 (363.88)	1617.46 (705.18)	2351.71 (1025.28)	3040.39 (1325.52)	3686.32 (1607.11)
31	14531.48 (6458.12)	931.34 (412.54)	1802.98 (798.72)	2618.76 (1160.22)	3382.24 (1498.62)	4096.79 (1815.4)
32	14363.72 (5964.92)	948.92 (395.22)	1835.15 (764.24)	2662.82 (1108.81)	3435.81 (1430.54)	4157.74 (1730.94)
33	15943.98 (6566.17)	1084.35 (445.45)	2094.94 (860.68)	3036.81 (1247.73)	3914.61 (1608.51)	4732.71 (1944.82)
34	15003.72 (5986.23)	1048.81 (419.27)	2024.31 (809.16)	2931.6 (1171.74)	3775.48 (1508.92)	4560.35 (1822.48)
35	17626.66 (6461.27)	1266.92 (465.34)	2442.78 (897.15)	3534.12 (1297.86)	4547.01 (1669.7)	5487.1 (2014.76)
36	17618.37 (6743.66)	1302.72 (495.82)	2509.12 (955.17)	3626.3 (1380.74)	4660.88 (1775.02)	5618.95 (2140.29)
37	19709.25 (7361.3)	1496.38 (559.86)	2879.14 (1077.12)	4156.91 (1555.03)	5337.67 (1996.59)	6428.78 (2404.56)
38	21501.94 (8800.23)	1670.43 (681.2)	3211.08 (1309.67)	4632.04 (1889.48)	5942.61 (2424.4)	7151.35 (2917.91)
39	19442.25 (6673.98)	1550.24 (534.05)	2976.87 (1025.35)	4289.74 (1477.32)	5497.92 (1893.12)	6609.76 (2275.64)
40	23422.18 (6876.62)	1915.65 (562.72)	3674.62 (1079.37)	5289.71 (1553.73)	6772.71 (1989.26)	8134.41 (2389.15)
41	23374.43 (10158.92)	1954.94 (850.02)	3746.36 (1628.9)	5387.96 (2342.61)	6892.25 (2996.59)	8270.73 (3595.85)
42	23911.37 (8280.15)	2045.15 (708.07)	3915.36 (1355.57)	5625.6 (1947.68)	7189.57 (2489.15)	8619.76 (2984.31)
43	28484.27 (12203.2)	2496.97 (1068.17)	4775.04 (2042.83)	6853.41 (2932.16)	8749.59 (3743.65)	10479.53 (4484.09)
44	25840.02 (9697.71)	2312.66 (871.14)	4418.33 (1664)	6335.54 (2385.64)	8081.15 (3042.44)	9670.54 (3640.24)
45	25144.25 (11502.83)	2293.28 (1051.03)	4377.4 (2006.01)	6271.43 (2873.72)	7992.71 (3662.15)	9556.99 (4378.53)
Total	17284.27 (8038.05)	1261.51 (688.22)	2429.68 (1316.55)	3511.5 (1890.3)	4513.45 (2414.27)	5441.51 (2892.86)

© 2024 Thorpe KE et al. *JAMA Network Open*.

eTable 6. Association between adult BMI and BMI of oldest child between ages of 11-20 years for adults with employer-sponsored insurance

F(46, 762) =	28.19
Prob > F =	0.0000
R-squared =	0.1391

adultbmi	Coefficient	Linearized std. err.	t	P>|t|	[95% conf.	interval]
**Child bmi**	**.2110344**	**.0152905**	**13.80**	**0.000**	**.1810205**	**.2410483**
female	.4332704	.1523782	2.84	0.005	.134166	.7323749
Race/ethnicity						
Non-Hisp Black	.5998529	.1877032	3.20	0.001	.2314087	.968297
Non-Hisp Other	-1.363107	.2886439	-4.72	0.000	-1.929689	-.796526
Hispanic	-.4513292	.1817663	-2.48	0.013	-.8081198	-.0945386
Age Categories						
35-44	-.2214038	.3281457	-0.67	0.500	-.8655236	.422716
45-54	-.4931513	.3516543	-1.40	0.161	-1.183416	.1971136
55-64	-1.234699	.4011934	-3.08	0.002	-2.022205	-.4471936
Education						
<HS_grad	-.9420832	.2205982	-4.27	0.000	-1.375097	-.5090693
Some_College	.0134969	.1886813	0.07	0.943	-.3568672	.3838609
College_grad	-.4466289	.1741736	-2.56	0.011	-.7885157	-.104742
Region						
midwest	.3572389	.2184968	1.63	0.102	-.0716502	.786128
south	.3274013	.1826765	1.79	0.073	-.0311759	.6859785
west	-.0169137	.2280486	-0.07	0.941	-.4645522	.4307247
married	.1719325	.2275022	0.76	0.450	-.2746333	.6184983
Number in Household						
Ages 0_5	.2413696	.2021943	1.19	0.233	-.1555192	.6382585
Ages 6_17	-.1386778	.0834375	-1.66	0.097	-.302458	.0251024
Ages 18_64	-.0807182	.1080725	-0.75	0.455	-.2928546	.1314182
Ages 65+	.6888594	.3483441	1.98	0.048	.005092	1.372627
Self report	.3998352	.1485034	2.69	0.007	.1083368	.6913337
year						
2002	.0090925	.2571031	0.04	0.972	-.4955773	.5137622
2003	-.1250422	.2732072	-0.46	0.647	-.6613228	.4112384
2004	.0964146	.2744335	0.35	0.725	-.4422731	.6351024
2005	.0724342	.2752349	0.26	0.792	-.4678267	.612695
2006	.3842723	.2714287	1.42	0.157	-.1485173	.9170618
2007	.5682648	.2781963	2.04	0.041	.0221911	1.114338
2008	.4714819	.2788902	1.69	0.091	-.075954	1.018918
2009	.1409372	.26144	0.54	0.590	-.3722454	.6541198
2010	.5115602	.2871475	1.78	0.075	-.0520839	1.075204
2011	.8849196	.2961323	2.99	0.003	.3036392	1.4662
2012	.8056203	.2992308	2.69	0.007	.2182578	1.392983
2013	1.040855	.3138878	3.32	0.001	.4247223	1.656988
2014	2.161602	.6002018	3.60	0.000	.9834608	3.339743
2015	1.159454	.3157909	3.67	0.000	.5395856	1.779322
2016	1.072785	.3169442	3.38	0.001	.4506532	1.694918
2018	2.078095	.3413002	6.09	0.000	1.408155	2.748036
2020	1.041621	.3535466	2.95	0.003	.3476419	1.735601
Child female	-.0396813	.1356334	-0.29	0.770	-.3059171	.2265545

© 2024 Thorpe KE et al. *JAMA Network Open*.

```
   Child age |  -.0064301   .0025424    -2.53   0.012    -.0114206   -.0014396
             |
 Family income as percent of poverty line
 100-199 FPL  |  -.2762436   .4757217    -0.58   0.562    -1.210042    .6575543
 200-399 FPL  |  -.0896552   .4679743    -0.19   0.848    -1.008246    .8289353
    400+ FPL  |  -.8638654   .4945581    -1.75   0.081    -1.834637    .1069067
             |
   Health status
   Very good  |    1.16369   .1536301     7.57   0.000     .8621284    1.465252
        good  |    2.43823   .1720202    14.17   0.000      2.10057     2.77589
        fair  |   3.788965   .2564246    14.78   0.000     3.285627    4.292303
        poor  |   4.359932   .5686905     7.67   0.000     3.243645    5.476219
             |
       _cons |   26.24239   .8123447    32.30   0.000     24.64783    27.83695
------------------------------------------------------------------------------
```

© 2024 Thorpe KE et al. *JAMA Network Open*.

eTable 7. Association between adult BMI and BMI of oldest child between ages of 11 to 45 years for adults with Medicare

F(46, 360)	=	8.72
Prob > F	=	0.0000
R-squared	=	0.1684

adultbmi	Coefficient	Linearized std. err.	t	P>|t|	[95% conf.	interval]
**Child bmi**	**.1849031**	**.0219198**	**8.44**	**0.000**	**.1418123**	**.2279939**
female	1.605667	.3519375	4.56	0.000	.9138149	2.29752
Race/ethnicity						
Non-Hisp Black	.4735777	.4458251	1.06	0.289	-.4028426	1.349998
Non-Hisp Other	-.8225257	.5151448	-1.60	0.111	-1.835217	.1901659
Hispanic	-.6146395	.4439384	-1.38	0.167	-1.487351	.2580719
Age Categories						
35-44	-.2766096	1.951944	-0.14	0.887	-4.113817	3.560598
45-54	-.8365612	1.990829	-0.42	0.675	-4.75021	3.077088
55-64	-.7556974	1.938713	-0.39	0.697	-4.566895	3.0555
65+	-2.240738	2.012319	-1.11	0.266	-6.196631	1.715156
Education						
<HS_grad	-.3957949	.3568459	-1.11	0.268	-1.097296	.3057065
Some_College	.4740029	.4189925	1.13	0.259	-.3496688	1.297675
College_grad	.4138589	.419801	0.99	0.325	-.4114021	1.23912
region						
Midwest	.9172772	.5505274	1.67	0.096	-.1649708	1.999525
South	.0942116	.3980972	0.24	0.813	-.6883833	.8768065
West	-.0478521	.48751	-0.10	0.922	-1.006218	.9105139
married	-.4547913	.4684905	-0.97	0.332	-1.375768	.4661855
Number in Household						
Ages 0_5	-.0555066	.4378862	-0.13	0.899	-.9163203	.805307
Ages 6_17	.5869486	.2058235	2.85	0.005	.1823328	.9915644
Ages 18_64	.0014037	.221645	0.01	0.995	-.4343146	.4371219
Ages 65+	.2618514	.3969694	0.66	0.510	-.5185263	1.042229
self report	.0449104	.3403241	0.13	0.895	-.6241119	.7139327
year						
2002	.0024558	.5791655	0.00	0.997	-1.13609	1.141002
2003	-.0586039	.6171058	-0.09	0.924	-1.271734	1.154526
2004	.6807274	.6370343	1.07	0.286	-.5715792	1.933034
2005	.6169068	.5925973	1.04	0.298	-.5480438	1.781857
2006	.9550216	.6372316	1.50	0.135	-.2976729	2.207716
2007	.5675834	.5889222	0.96	0.336	-.5901426	1.725309
2008	1.068688	.6539904	1.63	0.103	-.2169518	2.354327
2009	.1550229	.593	0.26	0.794	-1.010719	1.320765
2010	.9043524	.6847538	1.32	0.187	-.4417632	2.250468
2011	.5158884	.5914288	0.87	0.384	-.6467652	1.678542
2012	.4559333	.5627979	0.81	0.418	-.6504366	1.562303
2013	.751621	.5951698	1.26	0.207	-.4183868	1.921629
2014	-.0996889	.5299499	-0.19	0.851	-1.141485	.9421071
2015	.5303633	.6601905	0.80	0.422	-.7674648	1.828191
2016	.5167531	.6397396	0.81	0.420	-.7408717	1.774378
2018	-.1847806	.6398544	-0.29	0.773	-1.442631	1.07307

© 2024 Thorpe KE et al. *JAMA Network Open*.

```
 child female    |  -.4627601   .3121903    -1.48   0.139    -1.076476    .1509556
    Child age    |  -.0026822   .0015909    -1.69   0.093    -.0058097    .0004454
                 |
Family income as percent of poverty line
    100-199 FPL  |  -.4855613    .448229    -1.08   0.279    -1.366707    .3955845
    200-399 FPL  |  -.2058461   .4756191    -0.43   0.665    -1.140837    .7291444
       400+ FPL  |  -1.139787   .4722049    -2.41   0.016    -2.068066   -.2115085
                 |
    Health status
      Very good  |   .6577157       .388     1.70   0.091    -.1050297    1.420461
           good  |   1.961533   .3834682     5.12   0.000     1.207697     2.71537
           fair  |   2.435307   .4882331     4.99   0.000     1.475519    3.395094
           poor  |   2.879086   .5749293     5.01   0.000     1.748868    4.009304
                 |
           _cons |   27.01269   2.121343    12.73   0.000     22.84247    31.18291
------------------------------------------------------------------------------------
```

© 2024 Thorpe KE et al. *JAMA Network Open*.

eTable 8. Association between adult health care expenditures and BMI of oldest child between ages of 11 to 20 years for adults with employer-sponsored insurance

F(46, 762) =	9.73
Prob > F =	0.0000
R-squared =	0.0688

DV: Adult Health Exp	Coefficient	Linearized std. err.	t	P>|t|	[95% conf.	interval]
**Child bmi**	**41.31624**	**31.2988**	**1.32**	**0.187**	**-20.12042**	**102.7529**
female	1408.235	334.1757	4.21	0.000	752.279	2064.191
Race/ethnicity						
Non-Hisp Black	-1143.476	534.241	-2.14	0.033	-2192.142	-94.80975
Non-Hisp Other	-1049.678	551.932	-1.90	0.058	-2133.07	33.71366
Hispanic	-2265.219	418.7185	-5.41	0.000	-3087.124	-1443.313
Age categories						
35-44	-149.9787	945.0431	-0.16	0.874	-2005.011	1705.054
45-54	299.3877	943.8873	0.32	0.751	-1553.376	2152.152
55-64	1160.024	1062.756	1.09	0.275	-926.0688	3246.116
Education						
<HS_grad	-112.2744	634.6593	-0.18	0.860	-1358.052	1133.503
Some_College	986.8068	450.6237	2.19	0.029	102.2741	1871.34
College_grad	850.2486	432.7603	1.96	0.050	.7799345	1699.717
region						
midwest	357.0751	491.9662	0.73	0.468	-608.6092	1322.759
south	-708.8226	437.5535	-1.62	0.106	-1567.7	150.0547
west	-328.7995	485.0755	-0.68	0.498	-1280.958	623.359
married	1747.576	499.348	3.50	0.000	767.402	2727.75
Number in Household						
Ages 0_5	-567.5866	393.3565	-1.44	0.149	-1339.709	204.536
Ages 6_17	-423.1676	193.0869	-2.19	0.029	-802.1795	-44.15576
Ages 18_64	-165.3045	286.998	-0.58	0.565	-728.6551	398.0462
Ages 65+	-547.667	650.1126	-0.84	0.400	-1823.778	728.4442
Self report	516.7131	340.7855	1.52	0.130	-152.2176	1185.644
year						
2002	820.2609	422.4871	1.94	0.053	-9.042306	1649.564
2003	410.2485	422.6208	0.97	0.332	-419.3173	1239.814
2004	1672.321	621.5743	2.69	0.007	452.2283	2892.414
2005	590.8522	519.839	1.14	0.256	-429.544	1611.248
2006	1113.275	576.3101	1.93	0.054	-17.96828	2244.519
2007	1539.912	782.3274	1.97	0.049	4.275308	3075.549
2008	781.2011	611.3486	1.28	0.202	-418.8199	1981.222
2009	1360.906	519.949	2.62	0.009	340.2938	2381.518
2010	1881.907	625.6129	3.01	0.003	653.8867	3109.928
2011	919.4314	560.9756	1.64	0.102	-181.712	2020.575
2012	1053.336	605.3382	1.74	0.082	-134.8868	2241.56
2013	2627.787	752.3519	3.49	0.001	1150.989	4104.584
2014	2271.21	821.3016	2.77	0.006	659.0702	3883.349
2015	1081.284	770.366	1.40	0.161	-430.8737	2593.441
2016	3096.123	970.7103	3.19	0.001	1190.708	5001.538
2018	4553.84	1084.341	4.20	0.000	2425.379	6682.302
2020	4918.304	1782.221	2.76	0.006	1419.967	8416.64
Child female	562.5188	304.2377	1.85	0.065	-34.67189	1159.709
Child age	.4235402	7.273864	0.06	0.954	-13.85438	14.70147

© 2024 Thorpe KE et al. *JAMA Network Open*.

```
 Family income as percent of poverty line
 100-199 FPL  |  -559.2148   1827.409    -0.31   0.760    -4146.249     3027.82
 200-399 FPL  |  -1867.204    1732.49    -1.08   0.281    -5267.923    1533.514
    400+ FPL  |  -1796.175   1759.969    -1.02   0.308    -5250.833    1658.482
              |
 Health status
   very good  |    1059.13   273.1318     3.88   0.000     522.9978    1595.263
        good  |   3098.939   339.0841     9.14   0.000     2433.348     3764.53
        fair  |   8374.516   719.5907    11.64   0.000     6962.026    9787.007
        poor  |   18454.88   2338.087     7.89   0.000     13865.43    23044.33
              |
       _cons  |   1370.807   2220.249     0.62   0.537    -2987.337    5728.951
------------------------------------------------------------------------------
```

© 2024 Thorpe KE et al. *JAMA Network Open*.

eTable 9. Association between adult health care expenditures and BMI of oldest child between ages of 11 to 45 years for adults with Medicare

F(46, 360) = 5.40
Prob > F = 0.0000
R-squared = 0.0664

DV: Adult Hlth Exp	Coefficient	Linearized std. err.	t	P>|t|	[95% conf.	interval]
**Child bmi**	**97.05162**	**83.37189**	**1.16**	**0.245**	**-66.84406**	**260.9473**
female	-1335.013	1263.9	-1.06	0.291	-3819.638	1149.611
Race/ethnicity						
Non-Hisp Black	-1302.874	1644.7	-0.79	0.429	-4536.088	1930.341
Non-Hisp Other	-1311.826	2325.965	-0.56	0.573	-5884.299	3260.647
Hispanic	123.234	1579.808	0.08	0.938	-2982.413	3228.881
Age Categories						
35-44	4109.757	3495.429	1.18	0.240	-2761.692	10981.21
45-54	3850.761	3334.736	1.15	0.249	-2704.792	10406.31
55-64	6674.218	4807.123	1.39	0.166	-2775.811	16124.25
65+	396.4159	3665.896	0.11	0.914	-6810.145	7602.977
Education						
<HS_grad	-1468.104	1224.684	-1.20	0.231	-3875.635	939.4282
Some_College	3416.278	1524.96	2.24	0.026	418.4541	6414.103
College_grad	7967.68	4954.424	1.61	0.109	-1771.918	17707.28
region						
Midwest	-3208.965	3290.888	-0.98	0.330	-9678.321	3260.391
South	-4057.623	3360.769	-1.21	0.228	-10664.35	2549.107
West	-5386.37	3275.348	-1.64	0.101	-11825.18	1052.436
married	-2141.267	1448.677	-1.48	0.140	-4989.133	706.5993
Number in Household						
Ages 0_5	-3075.095	1336.162	-2.30	0.022	-5701.773	-448.4168
Ages 6_17	-323.37	731.1825	-0.44	0.659	-1760.757	1114.017
Ages 18_64	1131.479	1181.724	0.96	0.339	-1191.6	3454.558
Ages 65+	-1322.559	1336.677	-0.99	0.323	-3950.251	1305.133
self report	-1215.793	1698.827	-0.72	0.475	-4555.413	2123.828
year						
2002	62.08654	2988.655	0.02	0.983	-5813.127	5937.3
2003	9859.533	11836.56	0.83	0.405	-13409.24	33128.31
2004	-3578.742	2318.932	-1.54	0.124	-8137.389	979.904
2005	-3352.834	2726.575	-1.23	0.220	-8712.842	2007.173
2006	2300.521	3382.453	0.68	0.497	-4348.836	8949.878
2007	-634.2733	2739.524	-0.23	0.817	-6019.736	4751.189
2008	-1015.044	2688.985	-0.38	0.706	-6301.155	4271.068
2009	-1733.691	2541.898	-0.68	0.496	-6730.653	3263.271
2010	-776.9599	2515.645	-0.31	0.758	-5722.312	4168.392
2011	-2005.547	2526.839	-0.79	0.428	-6972.906	2961.811
2012	-2383.136	2503.162	-0.95	0.342	-7303.949	2537.676
2013	238.114	3131.498	0.08	0.939	-5917.906	6394.134
2014	308.6433	2740.127	0.11	0.910	-5078.005	5695.291
2015	-828.1276	2743.616	-0.30	0.763	-6221.635	4565.38
2016	-776.6498	2978.956	-0.26	0.794	-6632.798	5079.498
2018	136.9468	2923.818	0.05	0.963	-5610.808	5884.701
Child female	-494.5084	1186.23	-0.42	0.677	-2826.445	1837.428
Child age	6.399057	6.242771	1.03	0.306	-5.873224	18.67134

© 2024 Thorpe KE et al. *JAMA Network Open*.

```
                  |
 Family income as percent of poverty line
    100-199 FPL  |   671.8742   1748.227     0.38   0.701    -2764.859    4108.607
    200-399 FPL  |   -139.824   1835.525    -0.08   0.939     -3748.17    3468.522
       400+ FPL  |   1450.534   2185.036     0.66   0.507    -2844.894    5745.963
                  |
       Health status
      Very good  |    3187.79   1406.376     2.27   0.024     423.0819    5952.498
           good  |   6903.463   1536.141     4.49   0.000     3883.657    9923.269
           fair  |   13800.53    3005.49     4.59   0.000     7892.226    19708.84
           poor  |   17429.54   2124.308     8.20   0.000     13253.49    21605.59
                  |
           _cons |   3270.531   4289.903     0.76   0.446    -5162.727    11703.79
------------------------------------------------------------------------------------
```

© 2024 Thorpe KE et al. *JAMA Network Open*.

eTable 10. Patient characteristics by BMI of oldest child between ages of 11 to 20 years for adults with employer-sponsored insurance, Mean (std dev)

	CHILD BMI < 30	CHILD BMI >= 30
N	11782	1653
Health expenditures	6806 (14161)	7805 (16224)
BMI of oldest child (11-20) in family	22 (3.4)	34.2 (6.9)
Adult BMI	31.2 (5.2)	34.211 (6.588)
Sex		
male	53.2% (48.4%)	46% (52.1%)
female	46.8% (48.4%)	54% (52.1%)
Race/ethnicity		
NH White	74% (42.6%)	65.7% (49.6%)
NH Black	10.5% (29.7%)	15.7% (38%)
NH Other	5% (21%)	4.2% (21%)
Hispanic	10.6% (29.8%)	14.4% (36.6%)
Age Group		
20-34	4.1% (19.3%)	3.8% (20.1%)
35-44	37% (46.8%)	36.7% (50.4%)
45-54	47.1% (48.4%)	48.4% (52.2%)
55-64	11.8% (31.3%)	11% (32.7%)
Education		
< High school grad	5.5% (22.2%)	9.5% (30.6%)
High school grad	28.4% (43.8%)	33.8% (49.5%)
Some college	28.2% (43.6%)	32.8% (49.1%)
College grad	37.8% (47%)	23.9% (44.6%)
Census region		
Northeast	19.8% (38.6%)	14.7% (37%)
Midwest	25.3% (42.1%)	24.9% (45.2%)
South	35.7% (46.5%)	42.5% (51.7%)
West	19.3% (38.2%)	17.8% (40%)
Married		
no	12.5% (32.1%)	19.3% (41.2%)
yes	87.5% (32.1%)	80.7% (41.2%)
Number of household members aged 0-5	0.1 (0.3)	0.1 (0.4)
Number of household members aged 6-17	1.4 (0.9)	1.2 (1.1)
Number of household members aged 18-64	2.5 (0.8)	2.6 (1)
Number of household members aged 65+	0 (0.2)	0 (0.2)
Respondent for RU (self-report)		

© 2024 Thorpe KE et al. *JAMA Network Open*.

no	44.5% (48.2%)	40.6% (51.3%)
yes	55.5% (48.2%)	59.4% (51.3%)
Year		
2001	5.6% (22.3%)	5.3% (23.4%)
2002	6.0% (23.1%)	6.5% (25.8%)
2003	5.6% (22.2%)	5.9% (24.6%)
2004	5.8% (22.6%)	5.7% (24.3%)
2005	5.7% (22.6%)	6% (24.8%)
2006	5.6% (22.4%)	5.7% (24.2%)
2007	5.5% (22.1%)	4.5% (21.6%)
2008	6.1% (23.3%)	5.6% (24%)
2009	6.2% (23.5%)	7% (26.7%)
2010	6.5% (23.9%)	4.8% (22.4%)
2011	6.2% (23.3%)	5.9% (24.6%)
2012	6.1% (23.2%)	5.6% (23.9%)
2013	5.6% (22.3%)	4.7% (22%)
2014	5.6% (22.3%)	5.7% (24.2%)
2015	5.6% (22.3%)	6.7% (26.1%)
2016	5.1% (21.4%)	5.1% (23.1%)
2018	3.9% (18.8%)	5.8% (24.4%)
2020	3.3% (17.2%)	3.6% (19.5%)
Sex of oldest child (11-20) in family		
male	51.8% (48.5%)	56.1% (51.9%)
female	48.2% (48.5%)	43.9% (51.9%)
Age of oldest child (11-20) in family (months)	193.6 (32.1)	207.3 (28.8)
Family income as percent of poverty line		
<100 FPL	1.5% (11.7%)	2.1% (15%)
100-199 FPL	9.1% (27.8%)	14.3% (36.6%)
200-399 FPL	35% (46.3%)	42.5% (51.7%)
400+ FPL	54.5% (48.3%)	41.1% (51.4%)
Perceived health status		
excellent	15.6% (35.2%)	11.8% (33.7%)
very good	37% (46.8%)	29.6% (47.7%)
good	35.9% (46.5%)	40.3% (51.3%)
fair	9.4% (28.4%)	14.6% (36.9%)
poor	2.1% (13.9%)	3.7% (19.6%)

© 2024 Thorpe KE et al. *JAMA Network Open*.

eTable 11. Patient characteristics by BMI of oldest child between ages of 11 to 45 years for adults with Medicare, Mean (std dev)

	CHILD BMI < 30	CHILD BMI >= 30
N	2509	1265
Health expenditures	14953 (33022)	16675 (26847)
BMI of oldest child (11-20) in family	23.8 (3.4)	36.7 (6.6)
Adult BMI	31.7 (5.9)	34 (6.9)
Sex		
male	51.9% (49.2%)	44.7% (50.2%)
female	48.1% (49.2%)	55.3% (50.2%)
Race/ethnicity		
NH White	62.7% (47.6%)	58.4% (49.8%)
NH Black	16.4% (36.5%)	19.5% (40%)
NH Other	8% (26.8%)	5.7% (23.4%)
Hispanic	12.8% (32.9%)	16.4% (37.4%)
Age Group		
20-34	0.9% (9.1%)	0.4% (6.3%)
35-44	8.6% (27.6%)	6.2% (24.4%)
45-54	16.5% (36.6%)	13.2% (34.2%)
55-64	14.1% (34.3%)	16.4% (37.4%)
65+	59.9% (48.3%)	63.7% (48.6%)
Education		
< High school grad	23.4% (41.7%)	30.9% (46.7%)
High school grad	35.2% (47%)	35.5% (48.3%)
Some college	25.1% (42.7%)	21.6% (41.5%)
College grad	16.3% (36.4%)	12.1% (32.9%)
Census region		
Northeast	22.9% (41.4%)	19.1% (39.7%)
Midwest	19.7% (39.1%)	19.7% (40.2%)
South	38.7% (48%)	40.8% (49.6%)
West	18.7% (38.4%)	20.4% (40.7%)
Married		
no	35.8% (47.2%)	41.1% (49.7%)
yes	64.2% (47.2%)	58.9% (49.7%)
Number of household members aged 0-5	0.1 (0.3)	0.1 (0.3)
Number of household members aged 6-17	0.5 (0.9)	0.5 (0.8)
Number of household members aged 18-64	1.9 (1)	1.9 (1)
Number of household members aged 65+	0.9 (0.8)	0.9 (0.8)

© 2024 Thorpe KE et al. *JAMA Network Open*.

Respondent for RU (self-report)		
no	37.1% (47.6%)	40.4% (49.6%)
yes	62.9% (47.6%)	59.6% (49.6%)
Year		
2001	5.6% (22.7%)	4.4% (20.8%)
2002	5.4% (22.2%)	4.3% (20.5%)
2003	4.9% (21.4%)	5% (22%)
2004	4.2% (19.8%)	5.5% (23%)
2005	5% (21.4%)	4.4% (20.6%)
2006	4.5% (20.4%)	5.2% (22.4%)
2007	4.9% (21.3%)	5.8% (23.6%)
2008	5.4% (22.3%)	4.9% (21.9%)
2009	5.5% (22.6%)	7.5% (26.6%)
2010	6.6% (24.5%)	6.6% (25%)
2011	5.5% (22.4%)	5.5% (23%)
2012	6.2% (23.8%)	6.8% (25.5%)
2013	7.8% (26.5%)	7.4% (26.4%)
2014	8.5% (27.4%)	7.5% (26.6%)
2015	8.4% (27.3%)	7.3% (26.2%)
2016	6.3% (23.9%)	7.2% (26.1%)
2018	5.2% (21.8%)	4.8% (21.5%)
Sex of oldest child (11-45) in family		
male	60.4% (48.2%)	54.2% (50.3%)
female	39.6% (48.2%)	45.8% (50.3%)
Age of oldest child (11-45) in family (months)	351.7 (125.4)	388.4 (115.5)
Family income as percent of poverty line		
<100 FPL	12.6% (32.7%)	13.2% (34.2%)
100-199 FPL	24.1% (42.1%)	26.6% (44.6%)
200-399 FPL	32% (45.9%)	30.9% (46.6%)
400+ FPL	31.4% (45.7%)	29.3% (46%)
Perceived health status		
excellent	6.1% (23.6%)	4.1% (20.1%)
very good	18.6% (38.4%)	18.6% (39.3%)
good	36.3% (47.4%)	31% (46.7%)
fair	26.9% (43.7%)	30% (46.3%)
poor	12.1% (32.1%)	16.2% (37.2%)

© 2024 Thorpe KE et al. *JAMA Network Open*.
