# Supplementary material for: Estimated Reduction in Health Care Spending Associated With Weight Loss in Adults
Source: JAMA Netw Open. 2024 Dec 5;7(12):e2449200. doi: 10.1001/jamanetworkopen.2024.49200 (PMC11621981; doi:10.1001/jamanetworkopen.2024.49200)
Supplement: Supplement 2. — Data Sharing Statement [file jamanetwopen-e2449200-s002.pdf]

**Data Sharing Statement**

**Data**
**Data available:** Yes
**Data types:** Data (not involving human participants)
**How to access data:** request from author. All data are available online from the Medicare Expenditure Panel Survey-Household Component.
**When available:** With publication

**Supporting Documents**
**Document types:** None

**Additional Information**
**Who can access the data:** researchers whose proposed use have been approved
**Types of analyses:** any
**Mechanisms of data availability:** after approval of proposal
**Any additional restrictions:** none
